# Brazilian Research in Intensive Care Network (BRICNet): shaping the landscape of critical care research in Brazil and beyond

**DOI:** 10.62675/2965-2774.20250284

**Published:** 2025-05-04

**Authors:** Juliana Carvalho Ferreira, Adriano José Pereira, Alexandre Biasi Cavalcanti, Antonio Paulo Nassar, Ary Serpa, Bruno Adler Maccagnan Pinheiro Besen, Bruno Martins Tomazini, Cassiano Teixeira, Felipe Dal-Pizzol, Fernando Augusto Bozza, Fernando Godinho Zampieri, Flávia Ribeiro Machado, Glauco Adrieno Westphal, Israel Silva Maia, Leandro Utino Taniguchi, Luciano Cesar Pontes Azevedo, Marcio Soares, Otavio Ranzani, Pedro Vitale Mendes, Rafael Barberena Moraes, Regis Goulart Rosa, Rodrigo Santos Biondi, Suzana Margarete Lobo, Thiago Costa Lisboa, Viviane Cordeiro Veiga, Wagner Luis Nedel, Jorge Ibrain Figueira Salluh, * COLLABORATOR authors for the BRICNet Scientific Committe, André Miguel Japiassú, Bruno do Valle Pinheiro, Cássia Righy, Cíntia Magalhães Carvalho Grion, Eduardo Leite Vieira Costa, Fernando José da Silva Ramos, Flávio Geraldo Rezende de Freitas, João Gabriel Rosa Ramos, Luiz Marcelo Sá Malbouisson, Márcio Manozzo Boniatti, Pedro Kurtz, Roberta Muriel Longo Roepke, Thiago Domingos Corrêa, Vandack Alencar Nobre

**Affiliations:** 1 Universidade de São Paulo Faculdade de Medicina Hospital das Clínicas São Paulo SP Brazil Division of Pulmonology, Instituto do Coração, Hospital das Clínicas, Faculdade de Medicina, Universidade de São Paulo - São Paulo (SP), Brazil.; 2 Brazilian Research in Intensive Care Network São Paulo SP Brazil Brazilian Research in Intensive Care Network (BRICNet) - São Paulo (SP), Brazil.; 3 Hospital Israelita Albert Einstein Intensive Care Unit and Telemedicine São Paulo SP Brazil Intensive Care Unit and Telemedicine, Hospital Israelita Albert Einstein - São Paulo (SP), Brazil.; 4 HCor-Hospital do Coração HCor Research Institute São Paulo SP Brazil HCor Research Institute, HCor-Hospital do Coração - São Paulo (SP), Brazil.; 5 Intensive Care Unit A. C. Camargo Cancer Center São Paulo SP Brazil Intensive Care Unit, A. C. Camargo Cancer Center - São Paulo (SP), Brazil.; 6 Hospital Israelita Albert Einstein Intensive Care Unit São Paulo SP Brazil Intensive Care Unit, Hospital Israelita Albert Einstein - São Paulo (SP), Brazil.; 7 Monash University School of Public Health and Preventive Medicine Australian and New Zealand Intensive Care Research Centre Melbourne Australia Australian and New Zealand Intensive Care Research Centre, School of Public Health and Preventive Medicine, Monash University - Melbourne, Australia; 8 Universidade de São Paulo Faculdade de Medicina Hospital das Clínicas São Paulo SP Brazil Intensive Care Unit, Hospital das Clínicas, Faculdade de Medicina, Universidade de São Paulo - São Paulo (SP), Brazil.; 9 Hospital Sírio-Libanês Research and Education Institute São Paulo SP Brazil Research and Education Institute, Hospital Sírio-Libanês - São Paulo (SP), Brazil.; 10 Universidade Federal de Ciências da Saúde de Porto Alegre Department of Internal Medicine Porto Alegre RS Brazil Department of Internal Medicine, Universidade Federal de Ciências da Saúde de Porto Alegre - Porto Alegre (RS), Brazil.; 11 Universidade do Extremo Sul Catarinense SC Brazil Laboratory of Experimental Pathophysiology, Postgraduate Program in Health Sciences, Health Sciences Unit, Universidade do Extremo Sul Catarinense -Criciúma (SC), Brazil.; 12 Instituto D’Or de Pesquisa e Ensino Rio de Janeiro RJ Brazil Instituto D’Or de Pesquisa e Ensino - Rio de Janeiro (RJ), Brazil.; 13 Instituto Nacional de Infectologia Evandro Chagas Rio de Janeiro RJ Brazil Instituto Nacional de Infectologia Evandro Chagas, Fundação Oswaldo Cruz - Rio de Janeiro (RJ), Brazil.; 14 University of Alberta Faculty of Medicine and Dentistry Edmonton Canada Faculty of Medicine and Dentistry, University of Alberta - Edmonton, Canada.; 15 Universidade Federal de São Paulo Escola Paulista de Medicina Hospital São Paulo São Paulo SP Brazil Intensive Care Department, Hospital São Paulo, Escola Paulista de Medicina, Universidade Federal de São Paulo - São Paulo (SP), Brazil.; 16 Centro Hospitalar Unimed Joinville Joinville SC Brazil Centro Hospitalar Unimed Joinville - Joinville (SC), Brazil.; 17 Hospital Nereu Ramos Florianópolis SC Brazil Hospital Nereu Ramos - Florianópolis (SC), Brazil.; 18 Universidade de São Paulo Faculdade de Medicina Hospital das Clínicas São Paulo SP Brazil Discipline of Emergency Medicine, Hospital das Clínicas, Faculdade de Medicina, Universidade de São Paulo - São Paulo (SP), Brazil.; 19 Barcelona Instituto for Global Health Barcelona Spain Barcelona Instituto for Global Health - Barcelona, Spain.; 20 Universidade Federal do Rio Grande do Sul Porto Alegre RS Brazil Postgraduate Program in Pulmonology, Universidade Federal do Rio Grande do Sul - Porto Alegre (RS), Brazil.; 21 Universidade Federal do Rio Grande do Sul Hospital das Clínicas de Porto Alegre Porto Alegre RS Brazil Intensive Care Unit, Hospital das Clínicas de Porto Alegre, Universidade Federal do Rio Grande do Sul - Porto Alegre (RS), Brazil.; 22 Hospital Moinhos de Vento Department of Internal Medicine Porto Alegre RS Brazil Department of Internal Medicine, Hospital Moinhos de Vento - Porto Alegre (RS), Brazil.; 23 Hospital Brasília Brasília DF Brazil Hospital Brasília - Brasília (DF), Brazil.; 24 Hospital de Base Faculdade de Medicina de São José do Rio Preto São José do Rio Preto SP Brazil Intensive Care Division, Hospital de Base, Faculdade de Medicina de São José do Rio Preto - São José do Rio Preto (SP), Brazil.; 25 Universidade Federal do Rio Grande do Sul Hospital de Clínicas de Porto Alegre Porto Alegre RS Brazil Postgraduate Program in Pulmonary Science, Hospital de Clínicas de Porto Alegre, Universidade Federal do Rio Grande do Sul - Porto Alegre (RS), Brazil.; 26 A Beneficência Portuguesa de São Paulo São Paulo SP Brazil BP - A Beneficência Portuguesa de São Paulo - São Paulo (SP), Brazil.; 27 Grupo Hospitalar Conceição Intensive Care Unit Porto Alegre RS Brazil Intensive Care Unit, Grupo Hospitalar Conceição - Porto Alegre (RS), Brazil.; 1 Instituto Nacional de Infectologia Evandro Chagas Fundação Oswaldo Cruz Rio de Janeiro RJ Brazil Instituto Nacional de Infectologia Evandro Chagas, Fundação Oswaldo Cruz - Rio de Janeiro (RJ), Brazil; 2 Universidade Federal de Juiz de Fora Faculdade de Medicina Department of Clinical Medicine, Hospital Universitário Juiz de Fora MG Brazil Department of Clinical Medicine, Hospital Universitário, Faculdade de Medicina, Universidade Federal de Juiz de Fora - Juiz de Fora (MG), Brazil; 3 Instituto Estadual do Cérebro Paulo Niemeyer Rio de Janeiro RJ Brazil Instituto Estadual do Cérebro Paulo Niemeyer - Rio de Janeiro (RJ), Brazil; 4 Universidade Estadual de Londrina Londrina PR Brazil Universidade Estadual de Londrina – Londrina (PR), Brazil; 5 Universidade de Sao Paulo Faculdade de Medicina Hospital das Clínicas São Paulo SP Brazil Division of Pulmonology, Instituto do Coração, Hospital das Clínicas, Faculdade de Medicina, Universidade de Sao Paulo - São Paulo (SP), Brazil; 6 Universidade Federal de São Paulo Department of Anesthesiology, Pain and Intensive Care São Paulo SP Brazil Department of Anesthesiology, Pain and Intensive Care, Universidade Federal de São Paulo - São Paulo (SP), Brazil; 7 Hospital SEPACO São Paulo SP Brazil Hospital SEPACO - São Paulo (SP), Brazil; 8 Clínica Florence Salvador BA Brazil Clínica Florence - Salvador (BA), Brazil; 9 Universidade de São Paulo Faculdade de Medicina Hospital das Clínicas São Paulo SP Brazil Division of Anesthesia, Hospital das Clínicas, Faculdade de Medicina, Universidade de São Paulo - São Paulo (SP), Brazil; 10 Hospital de Clínicas de Porto Alegre Department of Critical Care Porto Alegre RS Brazil Department of Critical Care, Hospital de Clínicas de Porto Alegre - Porto Alegre (RS), Brazil; 11 Instituto D’Or de Pesquisa e Ensino Department of Intensive Care Medicine Rio de Janeiro RJ Brazil Department of Intensive Care Medicine, Instituto D’Or de Pesquisa e Ensino - Rio de Janeiro (RJ), Brazil.; 12 Universidade de São Paulo Faculdade de Medicina Hospital das Clínicas São Paulo SP Brazil Department of Surgery, Hospital das Clínicas, Faculdade de Medicina, Universidade de São Paulo - São Paulo (SP), Brazil; 13 Hospital Israelita Albert Einstein Department of Intensive Care São Paulo SP Brazil Department of Intensive Care, Hospital Israelita Albert Einstein - São Paulo (SP), Brazil; 14 Universidade Federal de Minas Gerais Faculdade de Medicina Interdisciplinary Center for Research in Intensive Care Medicine Minas Gerais MG Brazil Interdisciplinary Center for Research in Intensive Care Medicine, Faculdade de Medicina, Universidade Federal de Minas Gerais - Minas Gerais (MG), Brazil

**Keywords:** Research design, Critical care, Critical illness, Global health, Developing countries, Sustainable growth, Brazil

## Abstract

Critical illnesses such as sepsis and acute respiratory distress syndrome lead to millions of deaths globally, with a higher burden in low- and middle-income countries. Conducting multicentric clinical studies is essential to help minimize the burden of critical illnesses, particularly in areas where their impact is greater. However, conducting large-scale multicentric studies is challenging, and most large multicentric studies in critical care are from high-income countries, which limits their relevance in other contexts. This highlights the need for collaborative research networks in low- and middle-income countries to better address local needs. The Brazilian Research in Intensive Care Network (BRICNet) was created by a group of intensivists and researchers in 2007 and is dedicated to being the leading organization in Brazil for conducting collaborative clinical research to improve care for critically ill patients. BRICNet focuses on investigator-initiated and collaborative studies relevant to global intensive care, with a special emphasis on Brazilian context. Its mission includes advancing research methodology, scientific writing, and conducting large-scale multicenter studies to fill knowledge gaps in critical care. Since its creation, the network has published 71 articles, including 15 randomized controlled trials and 14 observational studies, many of them in collaboration with major Brazilian institutions and international networks. This review aims to critically assess the achievements of BRICNet, highlighting its high-impact publications, international partnerships, and capacity building, which have significantly contributed to the field of intensive care. Looking ahead, we also identify barriers and solutions for sustainable growth.

## INTRODUCTION

Critical illnesses, including sepsis and severe acute respiratory distress syndrome, are responsible for millions of deaths worldwide.^( 1 , 2 )^ Clinical studies aimed at critical illnesses are fundamental to reducing this situation. However, including many patients in several centers is a complex task that involves the collaboration of several researchers and institutions. Therefore, intensive care research networks are essential for carrying out large studies that can help minimize the burden of critical illnesses. However, most intensive care studies still come from high-income countries,^( 3 )^ and the results may not be applicable in low- and middle-income countries (LMICs), where the impact of critical illnesses is more significant. Thus, collaborative research networks in LMICs are needed to carry out studies that reflect the needs and conditions of critically ill patients in these countries. This review aims to critically assess the achievements of Brazilian Research in Intensive Care Network (BRICNet), highlighting its high-impact publications, international partnerships, and capacity building, which have significantly contributed to the field of intensive care. Looking ahead, we also identify barriers and solutions for sustainable growth.

### The Brazilian Research in Intensive Care Network

The understanding that improving care practices—and, consequently, outcomes in intensive care medicine—depends on generating and practically applying robust scientific evidence was the driving force behind the creation of BRICNet (https://bricnet.com.br/). This idea, combined with recognizing that the epidemiology, patient profiles, and care practices in large, middle-income countries like Brazil have particular characteristics of geography, socio-demographics, and health system that affect critical care outcomes, helped shape the network's structure, vision, and mission.

BRICNet is an independent clinical research network focused on intensive care. Since its foundation, BRICNet's mission has been to conduct investigator-initiated collaborative studies relevant to global intensive care medicine, with a special emphasis on the Brazilian context.^( 4 )^ Additionally, the network is committed to advancing clinical research methodology and scientific writing among Brazilian intensivists. Recognizing the need for scientific evidence to improve care delivery and outcomes of critically ill patients, BRICNet focuses primarily on designing, organizing, and conducting large-scale multicenter studies. These studies aim to identify and address gaps in knowledge, which often arise due to the lack of solid evidence on specific topics, such as fluid management, treatment of ventilator-associated tracheobronchitis, or protective ventilation strategies.^( 5 – 7 )^ BRICNet is also committed to better understanding how evidence generated in different settings, like high-income countries, translates to reality in Brazil. Examples are studies on the attributable mortality of sepsis and the outcomes of critically ill cancer patients.^( 8 , 9 )^ Additionally, BRICNet runs quality improvement studies aimed at enhancing the translation of research knowledge into clinical practice, such as the implementation of checklists^( 10 )^ or extended family visits in intensive care units (ICUs).^( 11 )^

BRICNet had a crucial role in bringing together researchers during the coronavirus disease 2019 (COVID-19) pandemic to focus on critical clinical issues of that period. Without BRICNet's support, such research would not have been possible in the Brazilian context within such a short timeframe.^( 12 – 14 )^

A central mission of BRICNet is to foster collaborative research. To achieve this goal, the network collaborates with leading research institutions in Brazil, including *Hospital do Coração* , *Hospital S* í *rio-Liban* ê *s* , *Hospital Israelita Albert Einstein* , *BP - A Beneficência Portuguesa de São Paulo* , *Hospital Moinhos de Vento* , *Hospital Alemão Oswaldo Cruz* and *Instituto Latino Americano de Sepse* . BRICNet has also partnered with the COVID-19 Brazil Coalition, the *Conselho Nacional de Desenvolvimento Científico e Tecnológico* (CNPq), the *Coordenação de Aperfeiçoamento de Pessoal de Nível Superior* (CAPES) the Brazilian Ministry of Science and Technology (MCTI, acronym from the Portuguese *Ministério de Ciência e Tecnologia* ), the *Fundação de Amparo à Pesquisa do Estado de São Paulo* (FAPESP), and pharmaceutical and medical organizations.

Additionally, BRICNet prioritizes cooperation and integration with international networks.^( 5 , 15 )^ Consequently, studies in collaboration with other research networks are performed and help build critical mass and research execution capacity. Since 2007, all these aspects evolved and matured and will be discussed in more detail in the following sections.

### The early years, in a nutshell

In 2007, nine investigators dreamed of leveraging the potential to promote collaborative critical care research among Brazilian centers by creating a research network. A study to evaluate the characteristics and outcomes of patients with cancer admitted to ICUs served as the embryo of this aim. BRICNet's denomination and logo were coined in the subsequent year, and the first informal investigators’ meeting was held during the Brazilian Congress of Intensive Care in Salvador, Bahia. In the same congress, the first survey of the network was widely promoted, leading to an understanding of the practice patterns of more than 1,000 intensivists on delirium and sedation.^( 16 )^ A multicentric study, including critically ill patients with cancer, was the first to collect patient data. It included 717 patients admitted to ICUs in 28 Brazilian hospitals, resulting in several publications.^( 9 , 17 – 21 )^ Subsequently, the Epidemiology of Respiratory Insufficiency in Critical Care (ERICC) study was performed by the network achieving a higher reach, including 773 patients admitted to 45 ICUs from 12 Brazilian states in 2011.^( 22 )^ The data from the ERICC study was subsequently explored in secondary analyses^( 23 , 24 )^ and included in larger studies of mechanical ventilation practices and outcomes of combined databases from several countries.^( 25 – 27 )^

The founding group recognized that for BRICNet to thrive and be able to conduct more studies in the long-term, it needed to draw inspiration from the successful trajectory of other critical care research networks, such as the Australian and New Zealand Intensive Care Society (ANZICS)^( 28 )^ and the Canadian Critical Care Trials Group (CCCTG).^( 29 )^ Therefore, in 2014, other well-established Brazilian investigators in the field were invited to join the group. In addition, a meeting with the expanded group of researchers took place in Búzios, Rio de Janeiro, to establish the basis to improve the network's structure, organization and governance. The group developed a new governance and strategic plan during the meeting, elected the first executive and scientific committees, and agreed on a roadmap for future activities. The process to prioritize questions is based on ample group discussion, during yearly strategic planning meetings and monthly Scientific Committee meetings, considering identified research gaps that can be addressed by our network, cost, complexity, potential for innovation and impact for patients.

### What has BRICNet produced so far?

BRICNet significantly contributed to the scientific community with high-quality research published in high-impact journals. Additionally, the network produced study protocols, statistical analysis plans, systematic reviews, narrative reviews, and secondary analyses of randomized clinical trials, and participated in clinical practice guidelines. The most frequent themes included acute respiratory failure,^( 22 )^ acute respiratory distress syndrome (ARDS),^( 30 )^ COVID-19,^( 12 )^ hemodynamics,^( 31 )^ sepsis,^( 8 )^ organizational aspects of intensive care,^( 32 )^ critical care in oncology,^( 17 , 19 )^ long-term outcomes of survivors of critical care,^( 33 , 34 )^ and management of potential organ donors.^( 35 , 36 )^

**Figure 1:**
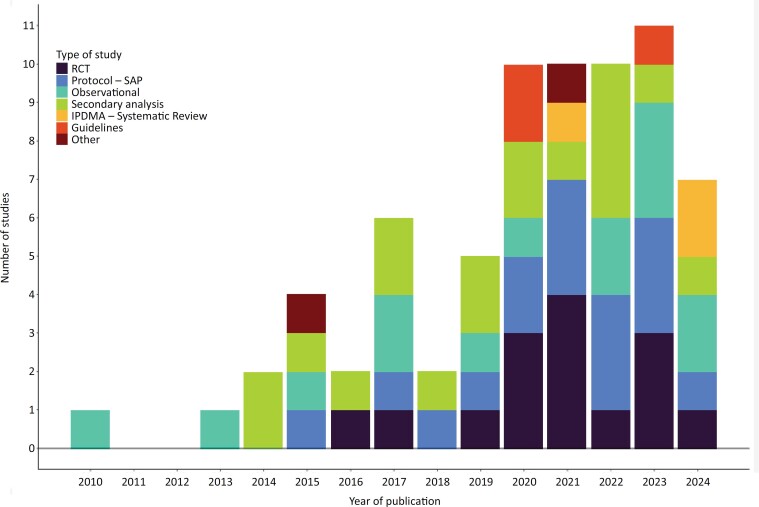
Publications from BRICNet and partners over time.

The network has published 71 articles distributed as follow ( Figure 1 ): 15 (21%) randomized controlled trials (RCTs), 14 (20%) observational studies, 16 (23%) protocols and statistical analysis plans, 18 (25%) secondary analysis of RCTs/observational studies, 3 (4%) clinical practice guidelines, 3 (4%) systematic reviews, and two others (3%).

RCT - randomized controlled trials; SAP - statistical analysis plan; IPDMA - individual participant data meta-analysis.

The total number of citations was 5,163, with an average of 73 citations overall and 220 citations for the RCTs ( Table 1S - Supplementary Material) . Eight (11%) are considered highly cited articles by Web of Science ( Table 1 ). The H-index of the network is 26. Many of them were presented in Hot-Topic sections in major meetings and received editorials.

**Table 1:** List of the ten most cited original clinical studies from BRICNet and partners

Study	Design	Population	Number of patients	Number of centers	Journal	Year	Citations
**COVID-19**							
CODEX *	RCT	ARDS COVID-19	299	41	JAMA	2020	825
COALITION I *	RCT	Hospitalized COVID-19	667	55	NEJM	2020	756
ACTION *	RCT	Hospitalized COVID-19 with high D-dimer	615	31	Lancet	2021	342
TOCIBRAS *	RCT	COVID-19 receiving oxygen or ventilated	129	9	BMJ	2021	294
**Respiratory failure**							
ART *	RCT	Moderate to severe ARDS	1,010	120	JAMA	2017	620
**Sepsis**							
SPREAD *	Observational	Sepsis patients in the ICU	2,632	227	Lancet Inf Dis	2017	178
**Cancer in the ICU**							
Cancer patients in the ICU *	Observational	Patients with cancer in the ICU	717	28	Crit Care Med	2010	254
**Fluid resuscitation**							
BASICS *	RCT-factorial	ICU patients requiring fluid expansion	11,052	75	JAMA	2021	147
**ICU organization**							
CHECK-LIST *	RCT-cluster	ICU patients	13,638	118	JAMA	2016	116
ICU Visits *	RCT-cluster cross-over	ICU patients/ families/ clinicians	1,685	36	JAMA	2019	99

Citations retrieved from the Web of Science Core Databases on September 5th, 2024.

* CODEX was funded by Coalition COVID-19 Brazil and *Aché Laboratórios Farmacêuticos* and coordinated by *Hospital Sirio-Libanês* ; COALITION I was funded by the Coalition Covid-19 Brazil and EMS Pharma; ART was funded by the *Programa de Desenvolvimento Institucional do Sistema Único de Saúde* (PROADI-SUS) from the Brazilian Ministry of Health and coordinated by *Instituto de Pesquisa HCor* ; ACTION was funded by Coalition COVID-19 Brazil and Bayer S.A., and coordinated by Academic Research Organization from *Hospital Israelita Albert Einstein* ; TOCIBRAS was funded by the hospitals and research institutes participating in the Coalition Covid-19 Brazil, Fleury Laboratory and *Instituto Votorantim* ; Cancer patients in the ICU was sponsored by *Instituto Nacional de Câncer* , Rio de Janeiro, Brazil and BRICNet; SPREAD was funded by *Fundação de Apoio a Pesquisa do Estado de São Paulo* (FAPESP); BASICs was funded by the Brazilian Ministry of Health through the *Programa de Desenvolvimento Institucional do Sistema Único de Saúde* (PROADI-SUS) and *Baxter Hospitalar* ; CHECKLIST was by PROADI from the Brazilian Ministry of Health, *the Agência Nacional de Vigilância Sanitária* (Anvisa), *Banco Nacional de Desenvolvimento Econômico e Social* (BNDES) and *Instituto D’Or de Pesquisa e Ensino* ; ICU visits was funded by the Brazilian Ministry of Health through the PROADI-SUS; ERICC was funded by *Hospital Sírio-Libanês* , São Paulo and the *Instituto D’Or de Ensino e Pesquisa* , Rio de Janeiro. RCT - randomized controlled trials; ARDS - acute respiratory distress syndrome; ICU - intensive care unit.

### Observational studies

In RCTs performed in ICUs there may be a high exclusion rate of research participants due to the potential delay in administering emergency therapies, especially due to the need for informed consent.^( 37 )^ Observational research in intensive care presents potential benefits, such as greater representation of the critically ill patient population in studies, enhanced by broader inclusion and fewer exclusion criteria compared to randomized controlled trials. Additionally, cohort studies are often time-efficient and cost-effective.^( 38 )^

BRICNet has successfully conducted large observational studies ( Table 1 ). The 12 observational studies have included more than 220,000 patients so far. Many of these studies have the potential to assist in the implementation of public health care policies, such as in the field of sepsis, both in the ICU and in emergency units.^( 8 , 39 )^ Furthermore, multicenter studies have evaluated quality of life and long-term survival after discharge from the ICU,^( 33 , 34 )^ an area of research with increasing relevance in the critical care scenario.

These are crucial research agendas, as low- and lower-middle-income countries bear a higher burden of critical illness than high-income countries.^( 40 )^ Regrettably, studies representing this population are scarce compared to those evaluating populations from high-income countries.^( 41 )^

### Randomized controlled trials

Over the past two decades, BRICNet has successfully conducted and published 15 randomized clinical trials in partnership with other institutions, which enrolled over 30,000 patients in 120 centers.

The patient populations studied include general ICU-admitted patients,^( 10 , 11 , 42 )^ patients with ARDS,^( 30 )^ hospitalized and non-hospitalized COVID-19 patients^( 12 , 14 , 43 – 47 )^ ICU patients requiring fluid resuscitation,^( 31 , 48 )^ and brain-dead patients.^( 35 )^ The types of studies conducted comprise three cluster-randomized trials,^( 10 , 11 , 35 )^ 11 individual and parallel-group randomized trials,^( 12 , 14 , 30 , 31 , 42 – 48 )^ one 2x2 factorial trial,^( 31 )^ and one adaptive multi-arm and multi-stage trial.^( 43 )^

The RCTs have been conducted across 12 countries. The median number of participating centers was 40.5 (interquartile range: 28.5 to 81.75), and the median number of participants was 1,191 (interquartile range: 307 to 9,525). The studies have resulted in publications in high-impact journals such as Journal of the American Medical Association (JAMA),^( 10 – 12 , 30 , 31 , 48 )^ JAMA Open,^( 35 )^ New England Journal of Medicine,^( 14 )^ The Lancet Regional Health,^( 43 , 47 )^ The Lancet,^( 46 )^ British Medical Journal,^( 45 )^ and Annals of the American Thoracic Society,^( 42 )^ with publication years ranging from 2016 to 2024. Five of these studies were accompanied by editorials in these journals,^( 11 , 12 , 30 , 31 , 48 )^ and six were recognized as hot topics at major conferences, including those of the European Society of Intensive Care Medicine and the Brazilian Congress of Intensive Care Medicine.^( 11 , 12 , 30 , 31 , 35 , 48 )^ Currently, there are four completed randomized trials under submission to journals (NCT03643939, NCT04972318, NCT01784159 and NCT05485051).

The Brazilian Ministry of Health, through the governmental Program to Support the Institutional Development of the Unified Health System (PROADI-SUS, acronym from the Portuguese *Programa de Apoio ao Desenvolvimento Institucional do Sistema Único de Saúde* ) has provided most of the funding for the studies conducted by BRICNet, in partnership with the research institutes of the hospitals that integrate this program and other governmental funding agencies.

### Guidelines

Incorporating evidence into critical care practice is widely recognized as a critical factor for the optimal care of critically ill patients, potentially enhancing patient and family outcomes and reducing healthcare costs.^( 49 )^ Thus, guidelines are valuable tools for synthesizing the best available evidence. During the COVID-19 pandemic, BRICNet participated in important guidelines for pharmacological interventions^( 50 )^ that played a critical role in supporting decisions regarding the pharmacological treatments of COVID-19 hospitalized patients. Developed in a task force that included representatives from the Brazilian Ministry of Health, national medical societies, and experts in guideline methodology, these guidelines were crucial in ensuring the appropriate treatment of patients. Similarly, a task force led by members of the BRICNet Scientific Committee, in association with medical societies, research institutions, and the General Coordination of the National Transplant System, developed guidelines for managing brain-dead potential organ donors.^( 36 )^ These recommendations are vital for this specific population, as structured, protocolized, and evidence-based management can enhance the benefits of interventions for many patients, optimizing the availability of organs for donation.

### BRICNet and capacity building

Since the network's inception, the BRICNet has had a Scientific Committee to provide shared decision-making on research and technical activities of the network. Some of the primary roles of the committee are to evaluate research proposals, foster projects under development, and promote scientific and educational meetings. The number of the Scientific Committee members has increased as the network participation in research activities grew. From the initial 11 members, it has grown to 40 members, including senior critical care researchers and young physicians starting their careers in critical care research. We developed objective and transparent criteria to invite new members to the Scientific Committee based on participation in BRICNet's clinical studies and academic achievements.

The network has 14 Associate Researchers and a fast and steady increase in membership in recent years, growing from 72 members in 2020 to 162 in 2024. Additionally, a mentorship research program has been created, in which members of the Scientific Committee guide young researchers. The project started in 2018 with one junior researcher and aims to stimulate new mentees to join. This program has already resulted in publications,^( 51 )^ master's dissertations, and doctoral theses. In recent years, BRICNet created a two-day training course, open to members and non-members, focusing on research methods and clinical studies, and more than a hundred students have completed the course.

BRICNet strongly advocates for collaboration and the inclusion of all investigators involved in a study. With a growing number of studies, the network has the challenge of expanding the number of ICUs able to recruit patients. ICUs are invited to participate in BRICNet studies through several strategies, from personal invitations to advertisements on our website and mailing lists. Some centers without previous participation in clinical research are now a major source of recruited patients in several studies. Since capacity building is one of our missions, we intentionally foster the inclusion of new centers by contacting any ICUs that demonstrate an interest in participating in a study for a meeting to identify if the ICU's structure meets the study's requirements and offer training for all aspects of the study and support for regulatory tasks, such as submission of protocol for ethical appreciation.

When an ICU is included as a center in BRICNet clinical study, the coordinating center trains the local team in the study procedures before initiation of recruitment through conferences, meetings, and in-person and/or online training sessions. It keeps regular contact to update centers of the study flow and offers support if necessary. Local researchers are trained to obtain informed consent, fill case report forms, deal with and report adverse events, collect high-quality data, and manage queries. In 2022, 162 investigators served as local principal investigators in BRICNet studies in ICUs distributed across Brazil. We acknowledge all participating centers, providing a certificate of being a BRICNet research center annually to recognize their involvement and create a sense of community. We reinforce among centers that engagement in research is associated with better healthcare performance and clinician understanding of research.^( 52 )^

### Challenges and solutions

To support BRICNet's sustainable growth, we faced several barriers and had to find targeted solutions to address each of them. The biggest challenge for any research network is securing funding. Over the years, BRICNet has used several strategies to overcome this barrier, from conducting low-cost observational studies, to partnerships with industry and healthcare organizations, particularly with the Ministry of Health through the PROADI program. Maintaining membership engagement over time can also be an important challenge, which we addressed by implementing regular communication, recognizing member contributions, and democratizing the Scientific Committee to allow more members to actively participate in study prioritization, propose new studies, and participate in the decisions. We also foster an environment of collegiality and collaboration by supporting shared decision-making and promoting the opportunity for the socialization of members. Finally, over the years, we faced the challenge of expanding the network with limited funding, which we overcame by formally recognizing the contributions of ICUs and its members and actively outreach to potential members in conferences and through periodic meetings open to anyone interested in the network.

### Strategic planning for a sustainable future

Over the years, BRICNet has successfully achieved its mission of leading multicenter studies with the potential to impact outcomes in Brazil and abroad, and several studies are ongoing.^( 5 , 6 , 53 – 55 )^

**Figure 2:**
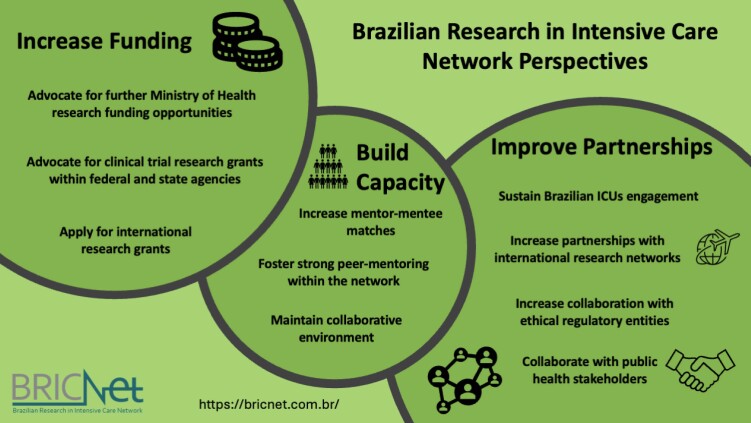
Perspectives for BRICNet.

Despite the enthusiasm for what has been accomplished so far, we must look forward to creating a sustainable future. Three intertwined core issues have been identified as crucial for a sustainable future ( Figure 2 ): funding, partnerships, and building capacity.

### Research funding

Being able to obtain regular funding is essential for sustaining research networks. It is necessary not only to cover the costs of multicenter trials but also to provide infrastructure to the network. In BRICNet, the operational costs of the network, including a secretary and costs for local meetings, come from revenue from the methodology course and annual fees paid by all members. None of the investigators receive any salary from BRICNet. Funding for each of the studies, which can be quite expressive, is sought and secured by the proposing investigators; BRICNet has taken advantage of the PROADI-SUS program, which has undoubtedly changed the landscape for funding multicenter research. This program can directly apply tax-waived resources from six Brazilian hospitals (defined by the Ministerial Ordinance as Superior Entities of Recognized Excellence) to public interest research after discussion with the Brazilian Ministry of Health stakeholders and others. This program has funded trials such as the BaSICS trial,^( 31 , 48 )^ the CHECKLIST^( 10 )^ and ICU visits trial^( 11 )^ and the ART trial.^( 30 )^ Collaboration between researchers from those hospitals, from other public and private hospitals and academic institutions has been at the core of capitalizing on this opportunity.

Nevertheless, with the network expansion and the identified need to address other research questions, the network is looking for additional funding opportunities in governmental and non-governmental agencies. The CNPq, CAPES, and FAPESP funded most Brazilian research from 2011 to 2018. However, grants from these institutions have not traditionally funded research personnel for conducting clinical trials beyond scholarships or have been unable to fund large, adequately powered, multicenter clinical trials. In recent years, BRICNet has started to work with these agencies to develop funding lines for multicenter clinical trials. Additionally, BRICNet has started to look for opportunities among international funding agencies.

Finally, beyond research funding, adopting innovative, more efficient trial designs (e.g., registry-based trials such as the IMPACTO-MR platform)^( 55 )^ will be valuable in reducing costs and streamlining further trials.

### Strategic partnerships

Partnerships are essential for a research network to endure and foster collaborative, multicenter research. The essential partnership to nurture is the connection of the large landscape of Brazilian ICUs with the research network. Without the participation of Brazilian ICUs in clinical trials, high-quality research output would not be possible so far and will not be possible in the future. More involvement of federal and philanthropic hospitals is also desired to streamline the inclusion of centers.

Partnerships may also go abroad. BRICNet has collaborated so far at various levels with ANZICS, CCCTG, the European Society of Intensive Care Medicine (ESICM), and the Latin American Intensive Care Network (LIVEN). We intend to keep and expand these partnerships to foster international collaboration and to advocate for more relevant global collaborations, with reciprocity and fair sharing of protagonism in international collaborations. Another aim includes the achievement of horizontal relationships between Latin-American centers and the Global North centers. Importantly, these partnerships need to be symmetrical regarding the work needed to gather desired data. Likewise, access to data for ancillary studies is also desirable, provided current ethical and regulatory requirements are respected.

One additional strategic partnership is the approximation of the network with regulatory authorities for the ethical conduct of multicenter trials in critical care. As previously recognized by ANZICS,^( 28 )^ alternative formats of consent may be needed to conduct adequately sized trials in critical care, such as delayed or opt-out consent, especially for testing interventions that represent variations in clinical practice and are therefore likely to be considered of minimal risk.^( 14 )^

### Building capacity

BRICNet is well positioned to foster mentorship for the next generation on the people side of the network's future. It is an important tool in shifting local power imbalances commonly observed in low- and middle-income countries through well-thought mentoring.^( 56 )^ Although BRICNet has established mentor-mentee relationships, the experience has been limited and needs to be expanded. This aspect becomes crucial in the Brazilian context, in which research career paths are less clear, opportunities are limited, and better work opportunities may attract talented researchers abroad and lead to brain drain.

As attempts to decolonize global health are made, a prolific scientific network from the Global South is at the right place at the right time to stand up for its mission and lead efforts in mentoring. To properly do so, the network must recognize the need to achieve a more equitable Scientific Committee in the future, including more women researchers and members of other underrepresented minorities in the Scientific Committee. Furthermore, the more horizontal nature of mature mentoring may foster a more collaborative research environment, essential for a virtuous cycle to improve research capacity and collaboration in LMICs. Finally, mentoring may benefit from transforming traditional mentoring by senior scientists to peer mentoring within the group, which may not only contribute to sustainability but also to a more flexible and sustainable mentoring model.^( 57 )^

In summary, this article reviews the achievements and ongoing efforts of BRICNet, a collaborative research network dedicated to improving critically ill patient outcomes in LMICs through investigator-led studies. Given the high burden of critical illness in LMICs, data produced by BRICNet hold significant potential to inform public health policies and improve patient care in Brazil and beyond. Since its creation, the network has published 71 articles, including 15 RCTs and 14 observational studies, addressing globally important topics in critical care. Alongside its research initiatives, BRICNet prioritizes capacity building by developing the research skills of its members and strengthening ICU practices nationwide. Over the years, the network has faced various challenges, including restricted funding availability, limited research infrastructure, and the need to expand member engagement. These barriers have been addressed through collective discussions to identify effective solutions. To ensure a sustainable future and continue to fulfill its mission, BRICNet will need ongoing support through multiple funding sources, strengthened partnerships, and ongoing capacity building.
