# Supplementary material for: Brazilian Research in Intensive Care Network (BRICNet): shaping the landscape of critical care research in Brazil and beyond
Source: Crit Care Sci. 2025 May 4;37:e20250284. doi: 10.62675/2965-2774.20250284 (PMC12266831; doi:10.62675/2965-2774.20250284)
Supplement: Supplementary file 1 [file 2965-2774-ccsci-37-e20250284-Mat-suppl.pdf]

Critical Care Science
https://doi.org/10.62675/2965-2774.20250284

SUPPLEMENTARY MATERIAL

Brazilian Research in Intensive Care Network (BRICNet): shaping the landscape of critical care research in Brazil and beyond

Juliana Carvalho Ferreira[1,2], Adriano José Pereira[2,3], Alexandre Biasi Cavalcanti[2,4], Antonio Paulo Nassar Junior[2,5], Ary Serpa Neto[2,6,7], Bruno Adler Maccagnan Pinheiro Besen[2,8], Bruno Martins Tomazini[2,9], Cassiano Teixeira[2,10], Felipe Dal-Pizzol[2,11], Fernando Augusto Bozza[2,12,13], Fernando Godinho Zampieri[2,14], Flávia Ribeiro Machado[2,15], Glauco Adrieno Westphal[2,16], Israel Silva Maia[2,17], Leandro Utino Taniguchi[2,9,18], Luciano Cesar Pontes Azevedo[2,6], Marcio Soares[12], Otavio Ranzani[1,2,19], Pedro Vitale Mendes[2,8], Rafael Barberena Moraes[2,20,21], Regis Goulart Rosa[2,22], Rodrigo Santos Biondi[2,23], Suzana Margarete Lobo[2,24], Thiago Costa Lisboa[2,25], Viviane Cordeiro Veiga[2,26], Wagner Luis Nedel[2,27], Jorge Ibrain Figueira Salluh[2,12], for the BRICNet Scientific Committee*

Table 1S - List of all the publications by BRICNet and partners

Study	DOI	Citations	Study design	Patients	Centers	Year	Funding
**COVID-19**							
Effect of dexamethasone on days alive and ventilator-free in patients with moderate or severe acute respiratory distress syndrome and COVID-19. The CoDEX randomized clinical trial	10.1001/jama.2020.17021	825	RCT	299	41	2020	Coalition COVID-19 Brazil and *Laboratórios Farmacêuticos Aché*
Hydroxychloroquine with or without azithromycin in mild-to-moderate COVID-19	10.1056/NEJMoa2019014	756	RCT	667	55	2020	Coalition Covid-19 Brazil and EMS Pharma
Therapeutic versus prophylactic anticoagulation for patients admitted to hospital with COVID-19 and elevated D-dimer concentration (ACTION): an open-label, multicentre, randomised, controlled trial	10.1016/S0140-6736(21)01203-4	342	RCT	615	31	2021	Coalition COVID-19 Brazil, Bayer SA.
Effect of tocilizumab on clinical outcomes at 15 days in patients with severe or critical coronavirus disease 2019: randomised controlled trial	10.1136/bmj.n84	294	RCT	129	9	2021	Coalition COVID-19 Brazil, Fleury Laboratory and *Instituto Votorantim*
Mortality outcomes with hydroxychloroquine and chloroquine in COVID-19 from an international collaborative meta-analysis of randomized trials	10.1038/s41467-021-22446-z	186	Systematic review	148		2021	No funding
Guidelines for the pharmacological treatment of COVID-19. The task-force/consensus guideline of the Brazilian Association of Intensive Care Medicine, the Brazilian Society of Infectious Diseases and the Brazilian Society of Pulmonology and Tisiology	10.5935/0103-507x.20200039	58	Guideline	N/A	N/A	2020	*Associação Hospitalar Moinhos de Vento*, *Hospital Alemão Oswaldo Cruz* and *Hospital Sírio-Libanês*
COVID-19-associated ARDS treated with DEXamethasone (CoDEX): study design and rationale for a randomized trial	10.5935/0103-507X.20200063	21	Protocol paper	N/A	N/A	2020	Coalition COVID-19 Brazil and *Laboratórios Farmacêuticos Aché*
Hydroxychloroquine versus placebo in the treatment of non-hospitalised patients with COVID-19 (COPE - Coalition V): A double-blind, multicentre, randomised, controlled trial	10.1016/j.lana.2022.100243	20	RCT	1,372	56	2022	No funding

Continue...

This is an open access article under the CC BY license (https://creativecommons.org/licenses/by/4.0/).

...continuation

Association between acute disease severity and one-year quality of life among post-hospitalisation COVID-19 patients: Coalition VII prospective cohort study	10.1007/s00134-022-06953-1	17	Observational	1,508	84	2023	Pfizer, EMS; *Laboratórios Farmacêuticos*; Bayer; Coalition COVID-19 Brazil, Fleury Laboratory and *Instituto Votorantim*
Randomized clinical trial to evaluate a routine full anticoagulation strategy in patients with coronavirus infection (SARS-CoV2) admitted to hospital: Rationale and design of the ACTION (AntiCoagulaTIon cOroNavirus)–Coalition IV trial	10.1016/j.ahj.2021.04.005	15	Protocol paper	600	40	2021	No funding
Rationale and design of tocilizumab in patients with moderate to severe COVID-19: an open label multicentre randomized controlled trial (TOCIBRAS)	10.5935/0103-507X.20200060	13	Protocol paper	N/A	9	2020	Coalition COVID-19 Brazil and *Laboratórios Farmacêuticos Aché*
Rivaroxaban to prevent major clinical outcomes in non-hospitalized patients with COVID-19: the CARE - COALITION VIII randomized clinical trial	10.1016/j.eclinm.2023.102004	7	RCT	657	33	2023	Coalition COVID-19 Brazil and Bayer S.A.
The II Brazilian Guidelines for the pharmacological treatment of patients hospitalized with COVID-19 Joint Guidelines of the *Associação Brasileira de Medicina de Emergência*, *Associação de Medicina Intensiva Brasileira*, *Associação Médica Brasileira*, *Sociedade Brasileira de Angiologia e Cirurgia Vascular*, *Sociedade Brasileira de Infectologia*, *Sociedade Brasileira de Pneumologia e Tisiologia* and *Sociedade Brasileira de Reumatologia*	10.5935/2965-2774.20230136-en.	6	Guideline	N/A	N/A	2023	PROADI-SUS
Quality of life and long-term outcomes after hospitalization for COVID-19: Protocol for a prospective cohort study (Coalition VII)	10.5935/0103-507X.20210003	4	Protocol Paper	N/A	N/A	2021	No funding
Antivirals for adult patients hospitalized with SARS-CoV-2 infection: a randomized, phase II/III, multicentre, placebo-controlled, adaptive study, with multiple arms and stages. COALITION COVID-19 BRAZIL IX - REVOLUTIOn trial	10.1016/j.lana.2023.100466	3	RCT	255	35	2023	MCTI, CNPq, *Clamed Farmácias*, *Cia LatinoAmericana de Medicamentos*, *Ciser Fixadores* and GP2U TeleHealth Australia, HCor São Paulo, Blanver, *Farmoquímica*, *Farmanguinhos - Fiocruz*, Cogeplan/*Fiocruz* and *Fiotec*
Effect of virtual information on the satisfaction for decision-making among family members of critically ill COVID-19 patients	10.1007/s00134-021-06616-7	3	Observational	84	1	2022	
Cardiovascular safety of azithromycin in patients hospitalized with COVID-19: a prespecified pooled analysis of the COALITION I and COALITION II randomized clinical trials	10.1016/j.amjcard.2023.11.069.	1	Secondary analysis	1,114		2024	No funding
Antisense therapy to block the Kallikrein-kinin pathway in COVID-19: the ASKCOV randomized controlled trial	10.1016/j.jcrc.2024.154892	0	RCT	111	13	2024	Ionis Pharmaceuticals
Antivirals for adult patients hospitalized with SARS-CoV-2 infection: a randomized, phase II/III, multicenter, placebo-controlled, adaptive study, with multiple arms and stages. COALITION COVID-19 BRAZIL IX - REVOLUTIOn: protocol and statistical analysis plan	10.5935/0103-507X.20220002-pt.	0	Protocol paper	N/A	N/A	2022	No funding

Continue...

...continuation

Rationale and design of the COVID-19 outpatient prevention evaluation (COPE - Coalition V) randomized clinical trial: hydroxychloroquine vs. placebo in non-hospitalized patients	10.36660/abc.20210832	0	Protocol paper	N/A	N/A	2022	No funding
**Respiratory failure**							
Effect of lung recruitment and titrated positive end-expiratory pressure (PEEP) vs low PEEP on mortality in patients with acute respiratory distress syndrome: a randomized clinical trial	10.1001/jama.2017.14171	620	RCT	1010	120	2017	PROADI-SUS
Clinical outcomes of patients requiring ventilatory support in Brazilian intensive care units: a multicenter, prospective, cohort study	10.1186/cc12594	98	Observational	773	45	2013	No funding
Early sedation and clinical outcomes of mechanically ventilated patients: a prospective multicenter cohort study	10.1186/cc13995	97	Secondary analysis	773	45	2014	No funding
Using Bayesian methods to augment the interpretation of critical care trials. An overview of theory and example reanalysis of the alveolar recruitment for acute respiratory distress syndrome trial	10.1164/rccm.202006-2381CP	72	Secondary analysis	1,010	120	2021	No funding
Heterogeneous effects of alveolar recruitment in acute respiratory distress syndrome: a machine learning reanalysis of the alveolar recruitment for acute respiratory distress syndrome trial	10.1016/j.bja.2019.02.026	37	Secondary analysis	1,010	120	2019	No funding
High flow nasal catheter therapy versus non-invasive positive pressure ventilation in acute respiratory failure (RENOVATE trial): protocol and statistical analysis plan	10.51893/2022.1.OA8	2	Protocol paper	N/A	N/A	2023	PROADI-SUS
Prospective, randomized, controlled trial assessing the effects of a driving pressure–limiting strategy for patients with acute respiratory distress syndrome due to community-acquired pneumonia (STAMINA trial): protocol and statistical analysis plan	10.62675/2965-2774.20240210-en	0	Protocol paper	N/A	N/A	2024	No funding
**Sepsis**							
The epidemiology of sepsis in Brazilian intensive care units (the Sepsis PREvalence Assessment Database, SPREAD): an observational study	10.1016/S1473-3099(17)30322-5	178	Observational	2,632	227	2017	FAPESP
Sepsis-associated outcomes in critically ill patients with malignancies	10.1513/AnnalsATS.201501-046OC	60	Secondary analysis	238	28	2015	INCA
Sepsis in Brazilian emergency departments: a prospective multicenter observational study	10.1007/s11739-022-03179-3	3	Observational	331	154	2023	FAPESP and CNPq
Attributable mortality due to nosocomial sepsis in Brazilian hospitals: a case-control study	10.1186/s13613-023-01123-y	1	Observational	3,588	37	2023	PROADI-SUS
**ICU organization**							
Effect of a quality improvement intervention with daily round checklists, goal setting, and clinician prompting on mortality of critically ill patients: a randomized clinical trial	10.1001/jama.2016.3463	116	RCT	13,638	118	2016	PROADI-SUS
Effect of flexible family visitation on delirium among patients in the intensive care unit: the ICU visits randomized clinical trial	10.1001/jama.2019.8766	99	RCT	1,685	36	2019	PROADI-SUS

Continue...

...continuation

Organizational characteristics, outcomes, and resource use in 78 Brazilian intensive care units: the ORCHESTRA study	10.1007/s00134-015-4076-7	94	Observational	59,693	78	2015	No funding
Effects of organizational characteristics on outcomes and resource use in patients with cancer admitted to intensive care units	10.1200/JCO.2016.66.9549	69	Secondary analysis	9,946	70	2016	No funding
ICU staffing feature phenotypes and their relationship with patients' outcomes: an unsupervised machine learning analysis.	10.1007/s00134-019-05790-z	39	Observational	129,680	93	2019	No funding
External validation of SAPS 3 and MPM0-III scores in 48,816 patients from 72 Brazilian ICUs	doi: 10.1186/s13613-017-0276-3	18	Secondary analysis	48,816	50	2017	No funding
Organizational factors associated with target sedation on the first 48 h of mechanical ventilation: an analysis of checklist-ICU database	10.1186/s13054-019-2323-y	18	Secondary analysis	5,719	118	2019	No funding
Role of organizational factors on the 'weekend effect' in critically ill patients in Brazil: a retrospective cohort analysis	10.1136/bmjopen-2017-018541	16	Secondary analysis	59,693	78	2018	No funding
Study protocol to assess the effectiveness and safety of a flexible family visitation model for delirium prevention in adult intensive care units: a cluster-randomized, crossover trial (The ICU Visits Study)	10.1136/bmjopen-2017-021193	14	Protocol Paper	N/A	40	2018	No funding
Organizational factors associated with adherence to low tidal volume ventilation: a secondary analysis of the CHECKLIST-ICU database	10.1186/s13613-020-00687-3	11	Secondary Analysis	5,719	118	2020	No funding
Structure and process associated with the efficiency of intensive care units in low-resource settings: An analysis of the CHECKLIST-ICU trial database	10.1016/j.jcrc.2020.06.008	9	Secondary analysis	13,635	118	2020	No funding
A cluster randomized trial of a multifaceted quality improvement intervention in Brazilian intensive care units: study protocol	10.1186/s13012-014-0190-0	8	Protocol paper	N/A	N/A	2015	No funding
Comparing continuous versus categorical measures to assess and benchmark intensive care unit performance	10.1016/j.jcrc.2022.154063	5	Secondary analysis	719,317	128 Brazil and Uruguay, 83 Holanda	2022	CNPq, CAPES, FAPERJ, the *Pontifícia Universidade Católica do Rio de Janeiro*, *Instituto D'Or de Pesquisa e Educação*
Statistical analysis of a cluster-randomized clinical trial on adult general intensive care units in Brazil: TELE-critical care verSus usual Care On ICU PErformance (TELESCOPE) trial	10.5935/0103-507x.20220003-pt	2	Statistical analysis plan	N/A	N/A	2022	No funding
TELE-critical Care verSus usual Care On ICU PErformance (TELESCOPE): protocol for a cluster-randomised clinical trial on adult general ICUs in Brazil	10.1136/bmjopen-2020-042302	2	Protocol paper	N/A	N/A	2021	No funding
**Fluid repleacement**							
Effect of intravenous fluid treatment with a balanced solution vs 0.9% saline solution on mortality in critically ill patients: the BaSICS randomized clinical trial	10.1001/jama.2021.11684	147	RCT	11,052	75	2021	PROADI-SUS
Study protocol for the balanced solution versus saline in intensive care study (BaSICS): a factorial randomized trial	NCT02875873	41	Protocol paper	N/A	N/A	2017	No funding

Continue...

...continuation

Effect of slower vs faster intravenous fluid bolus rates on mortality in critically ill patients: the BaSICS randomized clinical trial	10.1001/jama.2021.11444	34	RCT	11,052	75	2021	PROADI-SUS
Association between type of fluid received prior to enrollment, type of admission, and effect of balanced crystalloid in critically ill adults: a secondary exploratory analysis of the BaSICS clinical trial	10.1164/rccm.202111-2484OC	26	Secondary analysis	10,520	75	2022	No funding
Conditional treatment effect analysis of two infusion rates for fluid challenges in critically ill patients: a secondary analysis of balanced solution versus saline in intensive care study (BaSICS) trial	10.1513/AnnalsATS.202211-946OC	8	Secondary analysis	10,465	75	2023	PROADI-SUS
Hierarchical endpoint analysis using win ratio in critical care: An exploration using the balanced solutions in intensive care study (BaSICS)	10.1016/j.jcrc.2022.154113	5	Secondary analysis	10,490	75	2022	PROADI-SUS
Effects of balanced solution on short-term outcomes in traumatic brain injury patients: a secondary analysis of the BaSICS randomized trial	10.5935/0103-507X.20220261-pt	1	Secondary analysis	10,490	75	2022	PROADI-SUS
Determinants of fluid use and the association between volume of fluid used and effect of balanced solutions on mortality in critically ill patients: a secondary analysis of the BaSICS trial	10.1007/s00134-023-07264-9	0	Secondary analysis	10,520	75	2024	No funding
**General ICU**							
Patterns of intravenous fluid resuscitation use in adult intensive care patients between 2007 and 2014: An international cross-sectional study	10.1371/journal.pone.0176292	84	Observational	6,707	426	2017	Baxter Healthcare, CSL Behring
The effects of performance status one week before hospital admission on the outcomes of critically ill patients	10.1007/s00134-016-4563-5	50	Secondary analysis	59,693	78	2017	No funding
An international comparison of the cost of fluid resuscitation therapies	10.1016/j.aucc.2020.06.001	9	Secondary analysis	N/A	187	2021	No funding
Balanced crystalloids versus saline for critically ill patients (BEST-Living): a systematic review and individual patient data meta-analysis	10.1016/S2213-2600(23)00417-4	4	Secondary analysis	34,685	75	2024	HCor/George Institute of Global Health
IMPACTO-MR: a Brazilian nationwide platform study to assess infections and multidrug resistance in intensive care units	10.5935/0103-507X.20220209-pt	4	Protocol paper	33,983	51	2023	No funding
Bundle of coated devices to reduce nosocomial infections in the intensive care unit. CRITIC pilot randomized controlled trial	10.1513/AnnalsATS.202003-206OC	2	RCT	118	12	2020	No funding
Protocol for balanced versus saline trialists: living systematic review and individual patient data meta-analysis of randomized controlled trials (BEST-Living study)	10.51893/2022.2.OA3	1	Protocol paper	N/A	N/A	2023	No funding
**Organ donors**							
Evidence-based checklist to delay cardiac arrest in brain-dead potential organ donors: the DONORS cluster randomized clinical trial	10.1001/jamanetworkopen.2023.46901	34	RCT	1,535	63	2023	PROADI-SUS

Continue...

...continuation

Brazilian guidelines for management of brain-dead donnors	10.1186/s13613-020-00787-0	10	Guidelines	N/A	N/A	2020	PROADI-SUS
DONORS (Donation Network to Optimize Organ Recovery Study): study protocol to evaluate the implementation of an evidence-based checklist for brain-dead potential organ donor management in intensive care units, a cluster randomized trial	10.1136/bmjopen-2018-028570	3	Protocol paper	N/A	N/A	2019	No funding
**Cancer in the ICU**							
Characteristics and outcomes of patients with cancer requiring admission to intensive care units: a prospective multicenter study	10.1097/CCM.0b013e3181c0349e	254	Observational	717	28	2010	No funding
Outcomes for patients with cancer admitted to the ICU requiring ventilatory support: results from a prospective multicenter study	10.1378/chest.13-1870	110	Secondary analysis	717	28	2014	No funding
**Renal failure**							
Association between piperacillin/tazobactam use and acute kidney injury in critically ill patients: a retrospective multicentre cohort study	10.1093/jac/dkae001	0	Observational	20,107	MIMIC, eICU database (US DATABASE)	2024	No funding
Long-term mortality and health-related quality of life after continuous versus intermittent renal replacement therapy in ICU survivors: a secondary analysis of the quality of life after ICU study	10.1177/08850666231224392	0	Observational	195	10	2024	PROADI-SUS
**Sedation and delirium**							
Delirium recognition and sedation practices in critically ill patients: a survey on the attitudes of 1015 Brazilian critical care physicians	10.1016/j.jcrc.2009.04.004	54	Survey	1,015	N/A	2015	No funding
**Other**							
Early and late mortality following discharge from the ICU: a multicenter prospective cohort study	10.1097/CCM.0000000000004024.	20	Observational	1,554	10	2020	PROADI-SUS
Prevalence and functional status of children with complex chronic conditions in Brazilian PICUs during the COVID-19 pandemic	10.1016/j.jped.2021.12.004	3	Observational	248	15	2022	No funding

RCT - randomized controlled trials; PROADI-SUS - *Programa de Desenvolvimento Institucional do Sistema Único de Saúde*; MCT - Ministry of Science and Technology; CNPq - *Conselho Nacional de Desenvolvimento Científico e Tecnológico*; Farmanguinhos - *Instituto de Tecnologia em Fármacos;* Fiocruz - *Fundação Oswaldo Cruz*; Cogeplan - *Coordenação Geral de Planejamento Estratégico;* Fiotec - *Fundação de Apoio à Fiocruz*; Inca - *Instituto Nacional do Cancer*; ICU – intensive care unit; FAPESP - *Fundação de Amparo à Pesquisa do Estado de São Paulo*; FAPERJ - *Fundação Carlos Chagas Filho de Amparo à Pesquisa do Estado do Rio de Janeiro*; HCor - *Hospital do Coração.*
